# Scabies in the age of increasing drug resistance

**DOI:** 10.1371/journal.pntd.0005920

**Published:** 2017-11-30

**Authors:** Samar Khalil, Ossama Abbas, Abdul Ghani Kibbi, Mazen Kurban

**Affiliations:** 1 Department of Dermatology, American University of Beirut, Beirut, Lebanon; 2 Department of Biochemistry and Molecular Genetics, American University of Beirut, Beirut, Lebanon; 3 Department of Dermatology, Columbia University Medical Center, New York, New York, United States of America; University of California San Diego School of Medicine, UNITED STATES

## Abstract

Scabies is an infestation of the skin by the mite *Sarcoptes scabiei*. It manifests with pruritic erythematous papules and excoriations, in addition to the pathognomonic burrows. Multiple drugs can be used for treatment, but resistance to conventional therapy is increasing throughout the years. This paper will review the mechanisms of resistance proposed in the literature and some of the potential solutions to this problem.

## Introduction

Scabies is an infestation of the skin by the mite *S*. *scabiei*. Transmission is by direct skin-to-skin contact or indirectly through fomites. Symptoms typically appear 3 to 6 weeks after an infestation. However, in patients with a previous exposure to the mite, symptoms can appear as early as 24 hours post exposure. Lesions consist of pruritic erythematous papules with excoriations. They’re usually symmetrical and involve the interdigital webs, the flexural aspect of wrists, the axillae, the peri-umbilical area, the elbows, the buttock, the feet, the genital area in males, and the peri-areolar area in females. The whole body—including the face and the scalp—can be involved in infants, the elderly, and immunocompromised individuals. The pathognomonic sign is the burrow, which represents the tunnel that the female mite digs to lay its eggs. Crusted scabies (CS) is a severe form that occurs in immunosuppressed individuals such as patients with acquired immune deficiency syndrome (AIDS). It manifests with extensive hyperkeratosis, mainly over the scalp and the extremities.

The diagnosis of scabies is usually clinical, but there are tools to help confirm it. The physician can perform skin scrapings or apply scotch tape to a burrow and observe the mite or its products under light microscopy. A biopsy taken at the site of a burrow may show the mite and its eggs [1]. On dermoscopy, the mite appears as a dark triangular shape (delta glider sign) [2].

Untreated scabies can lead to complications. The excoriated skin is a portal of entry to bacteria—mainly *Staphylococcus* and *Streptococcus*—leading to impetigo. During a scabies infestation, the skin microbiome of pigs is altered: there is a dramatic increase in *Staphylococcus*, with a shift from the commensal *Staphylococcus hominis* to the pathogenic *Staphylococcus chromogenes* [3]. In humans, these bacteria can also become invasive and lead to postinfectious complications such as poststreptococcal glomerulonephritis or rheumatic fever. Some studies show an increased risk of chronic kidney disease and bullous pemphigoid in patients with a previous history of scabies [4, 5].

### Search strategy and selection criteria

References for this review were identified through searches of PubMed for articles published from 1991 to 2017 by use of search terms such as “scabies,” “treatment,” “resistance,” “lindane,” “permethrin,” and “ivermectin.” Articles resulting from these searches and relevant references cited in those articles were reviewed. The most relevant articles were included in this review.

### Treatment

The primary goal in the management of scabies is to treat the patient successfully and to take all appropriate measures to control the transmission of the disease to other individuals. Patients and their close contacts should be treated, regardless of symptoms. Because the mean survival time of the mite outside of the host is around 48 to 72 hours, items used within the previous 3 days should be placed in a plastic bag for at least 72 hours. Clothes and bed linens should be washed in hot water (at least 60°C) and then machine dried [1].

Scabicides can be classified into topical and systemic agents (Table 1).

**Table 1:** Guidelines for the treatment of ordinary scabies.

Guidelines	Recommended Regimens	Alternatives
**CDC** **(Division of Sexually Transmitted Diseases Prevention, 2015) [13]**	• **Permethrin:** Apply once for 8 to 14 hours • **Ivermectin:** 200 mcg/kg on day 0 and 2 weeks later	• **Lindane:** Apply once for 8 hours
**European** **(World Health Organization/International Union against Sexually Transmitted Infections, 2010) [14]**	• **Permethrin:** Apply once for 8 to 12 hours • **Ivermectin:** 200 mcg/kg on day 0 and 2 weeks later • **Sulfur:** Apply for 3 consecutive days	• **Benzyl benzoate:** Apply for 2 to 3 consecutive days
**United Kingdom** **(Clinical Effectiveness Group, British Association for Sexual Health and HIV, 2016) [15]**	• **Permethrin:** Apply for 8 to 12 hours on day 0 and 1 week later • **Malathion:** Apply for 24 hours on day 0 and 1 week later	• **Ivermectin:** 200 mcg/kg on day 0 and 2 weeks later

Abbreviation: CDC, Centers for Disease Control and Prevention.

#### Topical

Topical agents are usually applied overnight over the entire body from the neck down. In infants and the elderly, they’re also applied to the face and the scalp.

- Permethrin: The 5% cream is typically used. In 1994, before the widespread use of permethrin, mites were killed within 1 hour of in vitro exposure to the drug. In the year 2000, 35% of mites from the same population remained alive after 3 hours [6]. Although there are no publications confirming clinical resistance in humans, there are several anecdotal reports, and there is a confirmed case of resistance in dogs [7].
- Lindane (gamma-benzene hexachloride): The 1% lotion or cream is used. Neurotoxicity may occur if it is systemically absorbed. Therefore, it is contraindicated in premature infants, patients with extensive skin disease—such as CS patients—and patients with uncontrolled seizure disorders. There are multiple reports of clinical resistance to lindane in humans [8, 9].
- Other remedies: Other options include crotamiton, precipitated sulfur, benzyl benzoate, and malathion.

#### Systemic

- Ivermectin: It is the only oral agent currently used for scabies. It is avoided in pregnancy and in children below 15 kg of weight. In 1997, mites grown in vitro in the presence of ivermectin survived for a mean time of 1 hour. In 2006, this time increased to 2 hours [10]. There are reports of clinical resistance in humans in the literature [11, 12].

Mass treatment is sometimes given to a whole community in a hyperendemic area to decrease the prevalence of the disease. This is certainly beneficial but can also contribute to resistance. In the Skin Health Intervention Fiji Trial (SHIFT), 2,051 participants from Fiji were randomly assigned to 3 different treatment groups. In the first group, called the standard care group, only affected individuals and their contacts were treated with permethrin; in the permethrin group, everyone applied permethrin; and in the ivermectin group, everyone received 1 dose of ivermectin. The primary outcome was the prevalence of scabies and impetigo after 1 year. The prevalence of scabies and impetigo declined by 49% and 32% in the standard care group, 62% and 54% in the permethrin group, and 94% and 67% in the ivermectin group, respectively [16].

### Resistance and its mechanisms

Persistent signs and symptoms after treatment can be due to several causes, including incorrect diagnosis, prescription of an inappropriate drug, noncompliance or incorrect application of the cream, local reaction to the cream, postscabetic reaction to the mite or its products, reinfection, delusions of parasitosis, and resistance [1].

Resistance to scabicides is increasing throughout the years. This constitutes a big problem because of the bothersome symptoms of an infestation, the high healthcare cost, the possible complications, and the associated social stigmatization. In 2013, scabies was added to the World Health Organization (WHO) list of neglected tropical diseases. This highlights the need for better research. Molecular studies on the mite have been restricted in the past. That is partly due to the fact that it is difficult to collect in large quantities; a typical infested human harbors, on average, around 5 to 15 mites [1]. Also, the mite does not survive long outside the host. In 2010, researchers in Queensland, Australia, succeeded in developing a porcine model of scabies that provides over 6,000 mites/g skin. These pigs were maintained on dexamethasone and developed crusts similar to those of CS patients [17].

In recent years, studies have identified 4 different players that could potentially contribute to scabicide resistance, as follows: (I) voltage-gated sodium channels, (II) glutathione S-transferase (GST), (III) ATP-binding cassette transporters, and (IV) ligand-gated chloride channels.

#### Voltage-gated sodium channels

**Fig 1:**
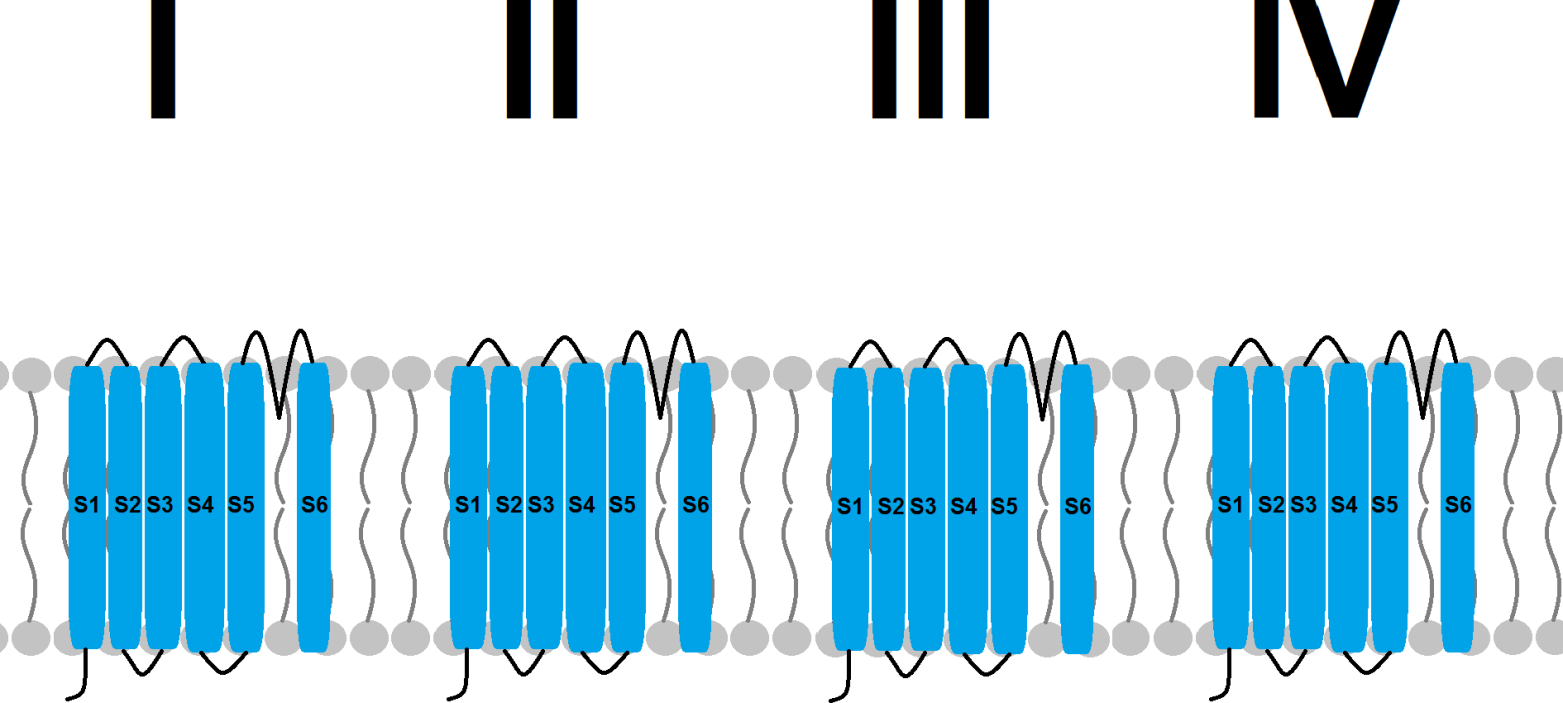
Structure of the voltage-gated sodium channel. The channel consists of 4 homologous domains (domains I to IV), with 6 transmembrane segments each (S1 to S6).

The voltage-gated sodium channel is essential for the normal functioning of neurons and myocytes. It normally consists of an alpha subunit associated with 1 or 2 beta subunits. The alpha subunit alone is sufficient for functioning. It is a complex of 4 homologous domains (I–IV). Each domain consists of 6 transmembrane segments (S1–S6). S5 and S6 form the pore, while S1–S4 are the voltage-sensitive part of the channel (Fig 1). Upon a change in the transmembrane voltage, these segments sense it and change their configuration to open the channel and allow sodium to enter the cell. Many drugs act by binding to receptors inside the pore, thereby blocking the flow of sodium. Local anesthetics and anti-epileptics are examples of such drugs. Other neurotoxins, such as permethrin, act by binding to sites away from the pore. An in vitro model of the channel suggests that the binding site of permethrin is located within a long hydrophobic cavity between the IIS4/IIS5 linker and the IIS5/IIIS6 helices. Upon binding to this site, the drug prevents the channel from closing. The resultant continuous sodium flow leads to repetitive firing of axons and excessive hyperactivity, placing the mite in what is called a “state of knockdown.” This state will be followed by paralysis and eventually death of the mite.

In several species, resistance to permethrin (also called knockdown resistance) is due to mutations in this sodium channel. These mutations do not always occur in the binding site of the drug. Permethrin preferentially binds to the channel when it is in an open or active state. Some mutations shift the channel to its closed state, thereby reducing the binding of the drug [18].

One article shows a mutation in the voltage-gated sodium channel leading to resistance in *S*. *scabiei* var. *Canis*. Mites were collected from laboratory dogs that had been maintained under permethrin treatment for many years and had become tolerant to therapy. Sequencing of the alpha subunit of the sodium channel revealed a guanine (G) to adenine (A) single nucleotide polymorphism within segment 6 of domain III, substituting aspartic acid for glycine. This mutation was not identified in dogs from the same population who did not develop clinical tolerance [19].

#### GST

**Fig 2:**
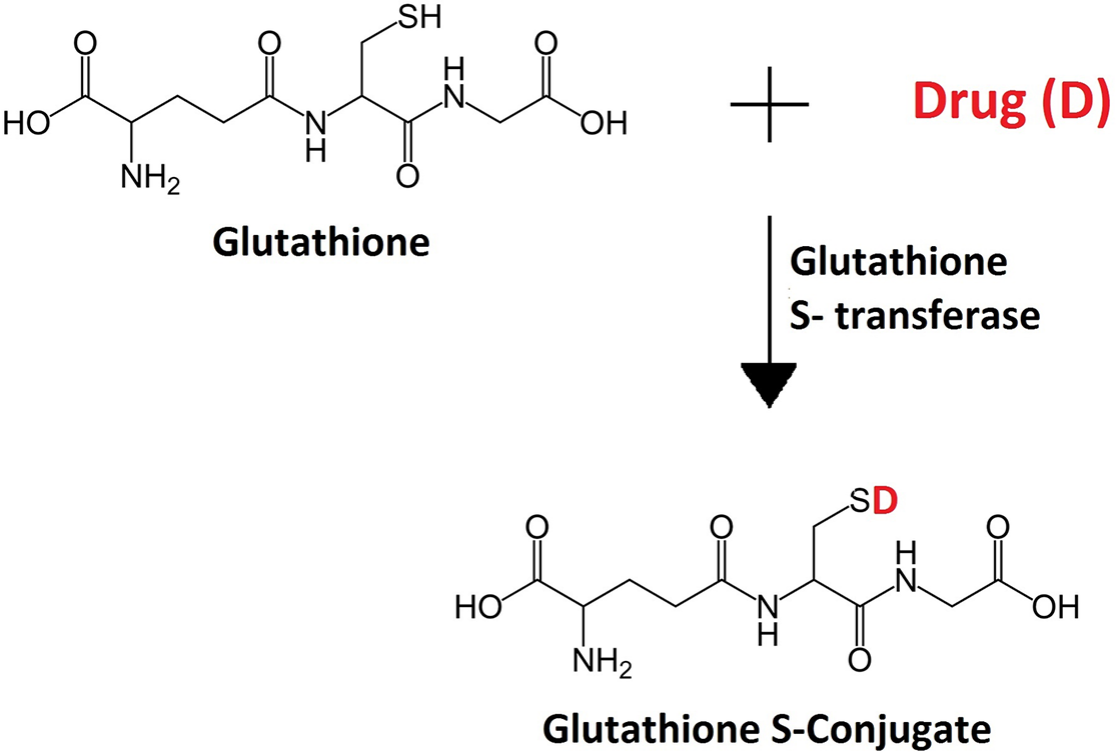
Reaction catalyzed by GST. GST catalyzes the formation of a thioester bond between reduced glutathione and the drug, forming a glutathione S conjugate of the drug. GST, glutathione S-transferase.

The enzyme GST catalyzes the formation of a thioester bond between reduced glutathione and drugs (Fig 2). This bond tags the drug for elimination from the body. Increased activity or expression of GST has been linked to resistance to both permethrin and ivermectin in different mite species.

In one study by Pasay et al., permethrin-naïve *S*. *scabiei* were isolated from pigs, tolerant mites from a human with recurrent CS, and resistant mites from dogs. In the tolerant and resistant mites, there was a statistically significant increase in GST activity, with the increase being higher in the resistant mites. Furthermore, the transcription of the enzyme was also significantly up-regulated in the resistant population. Mites collected from the same CS patient after ivermectin treatment also had higher transcription levels of GST compared with those collected prior to therapy. Therefore, GST might be mediating cross-resistance to both ivermectin and permethrin in scabies. However, the ivermectin molecule is considered too large to directly bind the GST active site, and there are currently no known glutathione conjugates of the drug. One hypothesis is that ivermectin does not bind to the active site of the enzyme but rather to a different site. In this way, the enzyme sequesters the drug and reduces its availability. Further studies are needed to confirm this hypothesis [7]. Another article shows an increase in the activity of 2 other metabolic enzymes in these resistant mites: the cytochrome p450 monooxygenase and esterase [20].

#### ATP-binding cassette transporters

**Fig 3:**
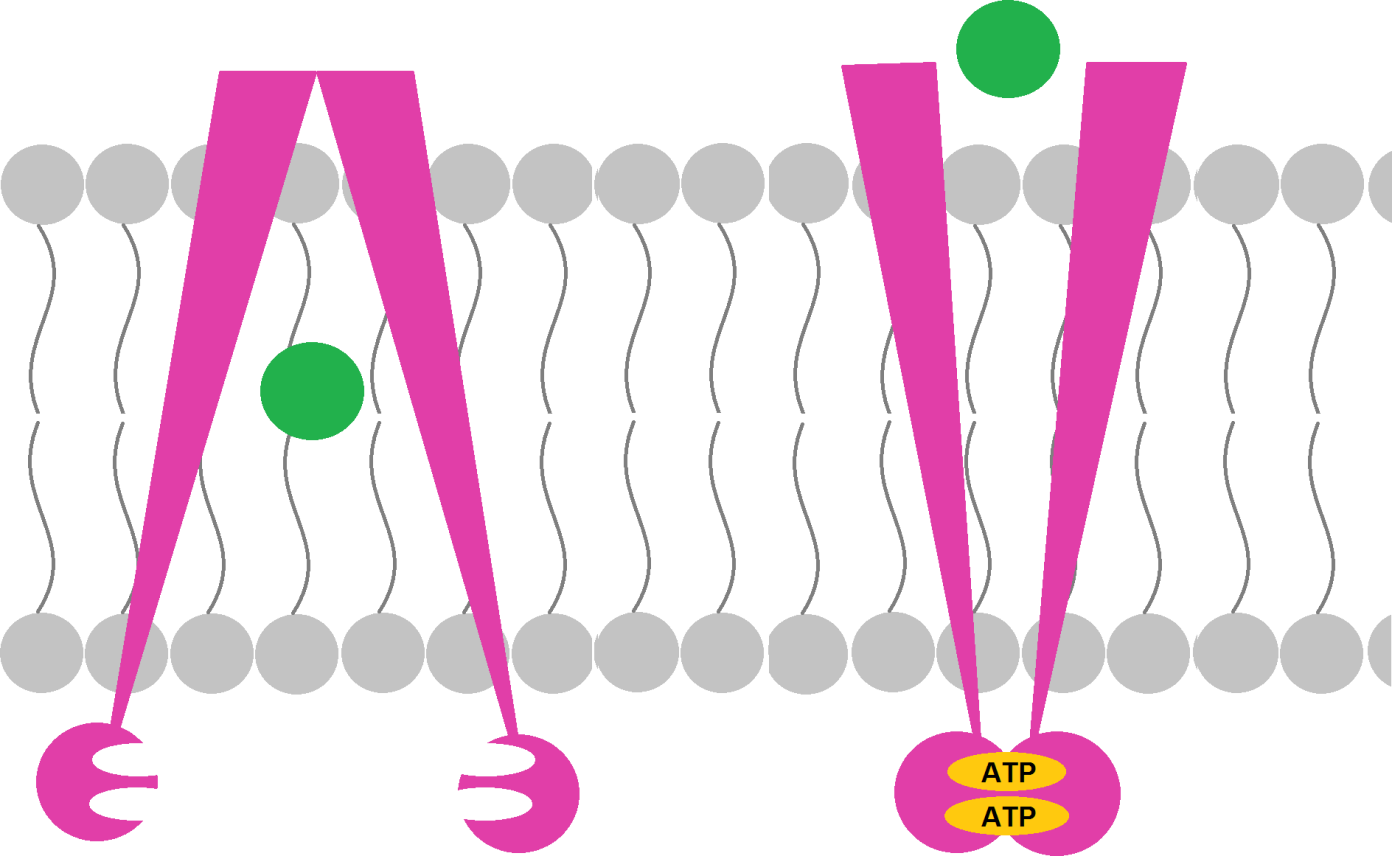
Mechanism of action of ABC transporters. Upon ATP binding, the transporter changes its configuration to allow the export of the drug to the extracellular space. ABC, ATP-binding cassette.

ATP-binding cassette (ABC) transporters function in either the uptake or the export of molecules. Like their name implies, they require energy from ATP to mediate the transport (Fig 3). They’re classified into 8 subfamilies (A to G), the most well-known of which is the B subfamily, also called the permeability glycoprotein (P-glycoprotein) or the multidrug-resistant protein (MDRP). Increased expression of MDRP is associated with resistance to several drugs, including chemotherapeutic agents and ivermectin.

In the study by Mounsey et al. previously mentioned in the GST section, the authors also studied the expression of MDRP in the mites of the CS patient. Its transcription was increased by almost 3-fold after a single dose of ivermectin [7]. There are no other studies on the expression of these transporters in resistant mites, but one article identified 9 different ABC transporters in *S*. *scabiei* [21].

#### Ligand-gated chloride channels

**Fig 4:**
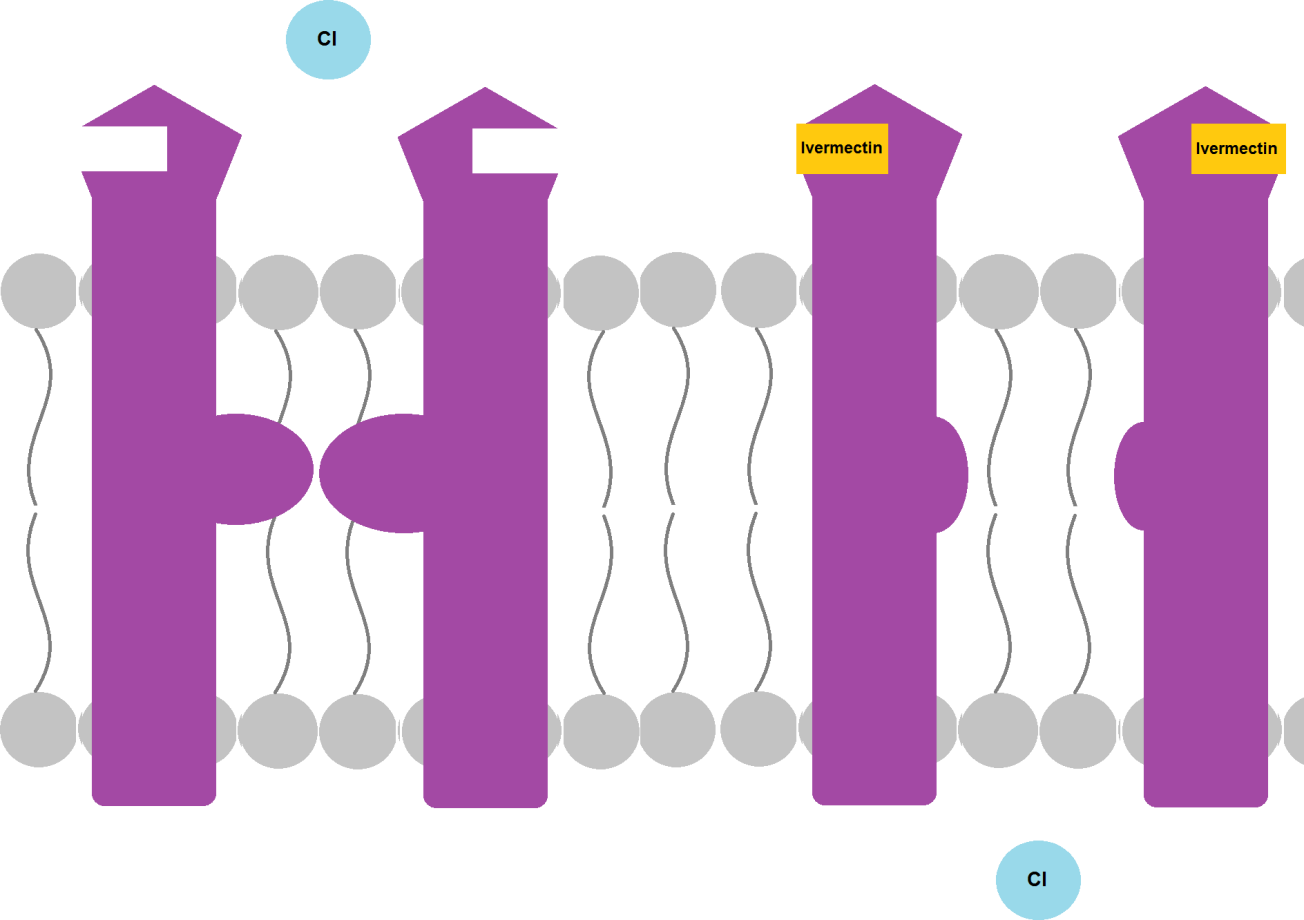
Mechanism of action of ivermectin on ligand-gated Cl channels. Upon drug binding, the pore of the channel opens, allowing Cl to enter the cell. These channels also respond to endogenous ligands such as GABA and glutamate. Cl, chloride.

Ligand-gated chloride channels are a superfamily of proteins that are essential for the functioning of neurons and muscles. Ligand binding results in a flow of chloride to the inside of the cell, leading to hyperpolarization. Ivermectin acts on these channels, mainly the glutamate and GABA-gated channels. Upon drug binding, the pore remains open, resulting in a continuous flow of chloride, paralysis, and death of the mite (Fig 4).

Mutations in this channel are found in several species that are resistant to ivermectin. No such mutations have been identified in scabies. However, one team identified and sequenced a chloride channel in scabies with a structure similar to ligand-gated channels. This channel did not respond to glutamate, gamma-aminobutyric acid (GABA), nor to other known ligands of such channels, but it was very sensitive to the extracellular pH. At pH less than 6, it was closed, and at a pH of 9, it had a maximal response. Ivermectin was found to activate this channel even at pH below 6, and the effect of the drug remained even when it was washed off the medium. The authors concluded that this channel might be playing a role in ivermectin resistance in scabies [22].

### Solutions

It is clear that resistance is increasing. Therefore, newer therapies or alternative control methods are needed. These can be classified into the following 4 sections: new drugs, insect growth regulators, natural products, and vaccination.

#### New drugs

Moxidectin (MOX) is a well-established treatment of scabies in animals like sheep and dogs. It is related to ivermectin and has the same mechanism of action, but there are important differences between the two. MOX is more lipophilic, leading to more tissue retention. Its mean half-life is more than 20 days, whereas that of ivermectin is around 14 hours. This is important because ivermectin does not have a strong ovicidal effect, and so a second dose is needed to kill any new larvae that have hatched from the remaining eggs. On the other hand, 1 dose of MOX can be retained in the skin through the whole 14-day life cycle of the mite. Therefore, 1 dose of this drug might be enough to eliminate the infestation. Another advantage of MOX is that it seems to prevent reinfection for a good duration of time. In 2 different experiments, the drug prevented the reinfection of sheep for up to 54 days. Finally, preliminary studies suggest that MOX is less toxic than ivermectin and is a poorer substrate for P-glycoproteins [23].

In a study by Bernigaud et al., 12 ivermectin-naïve pig models were randomly assigned to receive 1 dose of MOX, 2 doses of ivermectin, or placebo. By the end of the study, all the eggs were killed, and symptoms disappeared in the MOX-treated pigs. However, in the ivermectin group, eggs, symptoms, and immunoglobin (Ig)G levels persisted in some of the pigs. MOX achieved a plasma concentration almost 6 times higher than ivermectin and was detectable in the blood until the studyend (day 47). Ivermectin, on the other hand, lasted only until 7–9 and 12 days after the first and second doses, respectively. MOX also had more persistent levels in the skin [24].

Adjunct therapies that could be combined with traditional scabicides include permethrin and ivermectin synergists. These can counteract some of the mechanisms of resistance by inhibiting the metabolism or the export of scabicides [20, 25].

#### Insect growth regulators

Fluazuron is a compound used to control the growth of several animal ticks and fleas. It blocks the synthesis of chitin, the major constituent of the exoskeleton of arthropods such as scabies. It prevents the growth of new larvae within the eggs, but it does not have any effect on already formed mites. In one study, this drug was administered to 3 infested pigs. It resulted in a decrease in the number of juvenile stages and in the arrest of clinical progression of lesions for up to 5 weeks. Therefore, if combined with the traditional scabicides, this drug could potentially eliminate the need for a second dose [26].

#### Natural products

Tea tree oil derives from the plant *Melaleuca alternifolia*. It is routinely used as an adjunct therapy for scabies in the Royal Darwin Hospital in Australia. In an in vitro study, this plant-based product resulted in a shorter median survival time of the mites when compared to permethrin and ivermectin [27].

#### Vaccination

*S*. *scabiei* mites may develop new ways to evade newer therapies. Therefore, a radical solution, such as vaccination, is needed to eradicate them. A second scabies infestation is usually milder than the first, and there are many reports of animals being immune after a previous infestation. Therefore, a vaccine might be effective. Currently, the host immune response targeting the mite is not fully understood, but data suggest that a vaccine triggering a T helper type 1 (Th1) response is needed for protective immunity to occur.

Antibodies (IgG, IgM, and IgE) increase in both ordinary scabies (OS) and CS. This increase is higher in CS. Total IgA also increases in CS but decreases in OS. In one study, vaccination of goats with soluble proteins of *S*. *scabiei* resulted in high levels of scabies-specific IgG, but these goats were not protected from reinfestation. In another study, goats with a previous infestation had high specific IgG and IgE and were resistant to reinfestation. One might therefore assume that high specific IgE is an indicator of immunity, but CS patients have very high levels of IgE, and these patients do not have protective immunity [28].

As for the cellular response, CD4+ T cells are the dominant lymphocytes in the skin of OS patients. In CS, CD8+ T cells dominate. The fact that AIDS patients often develop CS suggests that CD4+ T cells are needed for protective immunity [28].

The mite also defends itself against the host’s immune system. For instance, serine proteases and serpins—found in the gut and feces of *S*. *scabiei*—were shown to inhibit all 3 complement pathways. In the gut, this could provide a defense mechanism against swallowed plasma. Outside the gut, this may play a role in weakening the immune system of the skin, thereby contributing to the increased bacterial infections during a scabies infestation [29]. In vitro studies have shown that these 2 families of proteins provide a favorable environment for the growth of *Streptococcus pyogenes* and *Staphylococcus aureus* even in the presence of complement [30, 31].

Currently, experiments are being conducted to develop the optimal scabies vaccine. Studies have shown that the sera of infested animals react with extracts from the house dust mite. Scabies antigens that are homologous to house dust mite proteins are therefore being tested for vaccination, but it will probably take years before an effective vaccine becomes available in the market [28].

**Key learning points**

- Scabies belongs to the World Health Organization list of tropical neglected diseases.
- Resistance to conventional therapy is increasing throughout the years.
- Mechanisms of resistance may involve 4 different players: voltage-gated sodium channels, GST, ATP-binding cassette transporters, and ligand-gated chloride channels.
- Potential solutions to the problem of resistance include newer drugs, insect growth regulators, natural products, and vaccination.
- It will probably take years before an effective scabies vaccine becomes available in the market.

**Top five papers**

1. Chosidow O. Clinical practices. Scabies. The New England journal of medicine. 2006;354(16):1718–27. doi: 10.1056/NEJMcp052784. PubMed PMID: 16625010.
2. Thomas J, Peterson GM, Walton SF, Carson CF, Naunton M, Baby KE. Scabies: an ancient global disease with a need for new therapies. BMC infectious diseases. 2015;15:250. doi: 10.1186/s12879-015-0983-z. PubMed PMID: 26123073; PubMed Central PMCID: PMC4487193.
3. Mounsey KE, Holt DC, McCarthy J, Currie BJ, Walton SF. Scabies: molecular perspectives and therapeutic implications in the face of emerging drug resistance. Future microbiology. 2008;3(1):57–66. doi: 10.2217/17460913.3.1.57. PubMed PMID: 18230034.
4. Pasay C, Arlian L, Morgan M, Vyszenski-Moher D, Rose A, Holt D, et al. High-resolution melt analysis for the detection of a mutation associated with permethrin resistance in a population of scabies mites. Medical and veterinary entomology. 2008;22(1):82–8. doi: 10.1111/j.1365-2915.2008.00716.x. PubMed PMID: 18380658.
5. Liu X, Walton S, Mounsey K. Vaccine against scabies: necessity and possibility. Parasitology. 2014;141(6):725–32. doi: 10.1017/S0031182013002047. PubMed PMID: 24476932.
